# Experiences of Children With Osteogenesis Imperfecta in the Co-design of the Interactive Assessment and Communication Tool Sisom OI: Secondary Analysis of Qualitative Design Sessions

**DOI:** 10.2196/22784

**Published:** 2021-08-10

**Authors:** Maia Siedlikowski, Lianna Curiale, Frank Rauch, Argerie Tsimicalis

**Affiliations:** 1 Ingram School of Nursing McGill University Montreal, QC Canada; 2 Shriners Hospitals for Children-Canada Montreal, QC Canada

**Keywords:** child health, symptom assessment, communication, mobile applications, software

## Abstract

**Background:**

Children with osteogenesis imperfecta (OI) experience a diversity of symptoms that expose them to difficult physical, mental, and social challenges. Sisom (DHealth) is an interactive assessment and communication tool designed to help children aged 6-12 years with chronic conditions express their symptoms. Recently, the co-design of the *Sisom OI* paper prototype was launched by seeking the perspectives of end users, including children with OI and their clinicians.

**Objective:**

The aim of this study is to describe the experiences that children with OI were prompted to share with researchers during the co-design of the *Sisom OI* paper prototype.

**Methods:**

A secondary analysis of qualitative data was conducted at a university-affiliated, pediatric, orthopedic hospital. The data sources consisted of interview transcripts, drawings, field notes, and observations derived from interviewing 12 children with OI who participated in the co-design of the *Sisom OI* paper prototype. The themes and subthemes identified from the data sources were generated using qualitative description.

**Results:**

Three themes were identified. The first, *Relating to Others*, described the balance between feeling different versus feeling similar to other children. The subthemes were *Common OI Experience*, *Feeling Different*, and *Feeling Just Like Others*. The second, *Relating to Their Condition*, described children’s positive and negative interactions with their own condition and health care. The subthemes were *Understanding Their Condition*, *Special Relationship with the Hospital*, and *Difficult Treatments and Procedures*. The third, *Reflecting on Capabilities*, described children’s recognition of their strengths and limitations. The subthemes were *Perceiving Limitations*, *Overcoming Isolation*, and *Celebrating Strengths*.

**Conclusions:**

This co-design process provided children with OI the space to not only contribute to the development of the end product but also eloquently describe their experiences. These findings, based on the descriptions given by the children themselves, offer us a unique understanding of what it means to grow up with OI.

## Introduction

### Background

Osteogenesis imperfecta (OI), otherwise known as brittle bones disease, is the most common inherited chronic bone disorder [1-4]. The prevalence of OI has been estimated at 1 in 13,500 and 1 in 9700 in 2 recent population-based studies from Scandinavia [5,6]. Its principal clinical feature is bone fragility, for which there is no cure. Thus, health services focus on the prevention and treatment of fractures to maximize mobility [7]. However, pain, fatigue, and varying degrees of physical limitations may hinder participation in daily activities and acceptance by peers and lead to feelings of fear, otherness, and isolation [4,8-13]. Relatively little attention has been directed toward understanding the day-to-day experiences of children with OI specifically and from their own perspective.

Children living with other chronic conditions have a unique understanding of their experience that cannot be conveyed through a proxy [14,15]. A growing body of knowledge demonstrates a gap between what children view as significant and what their parents perceive [15,16]. Children with chronic conditions are described as having an “intense embodied understanding of [their] disease” [17] and being “historians in their own right” [18]. As clinicians, we have a moral duty to elicit these children’s voices and partner with them to truly understand their perspective. This notion has been reinforced in the United Nations’ (1989) *Convention on the Rights of the Child*, which states that every child has the right to express themselves and be heard by others in matters that affect their well-being [19].

The concept of authentic listening is derived from the moral duty to treat children as competent moral agents [20]. It implies their active involvement in decision-making to help them foster a strong moral order and capacity in future decision-making. The concept of authentic listening goes beyond simply allowing a child to express himself/herself: it is about considering a child’s viewpoint as unique and valuable rather than immature and underdeveloped. Research that seeks to elicit the voices of children and include them as experts in decision-making is thus of great importance.

**Figure 1:**
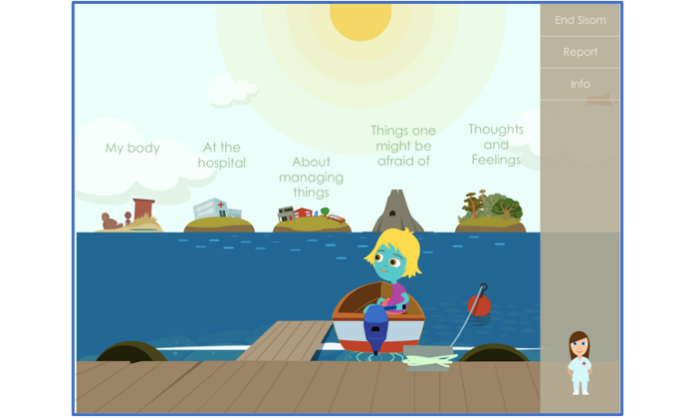
The child-created avatar has the option to travel to 1 of the 5 islands depicted in Sisom with the help of the navigator, Mary.

One strategy to elicit a child’s voice is to transform a child’s *place* (a place for children that is run by adults and their rules) into a child’s *space* (a place where children can take the lead) [21]. Efforts to create this *space* were made during the participatory co-design of a paper prototype called *Sisom OI* [13]. This was the first step our research team embarked on to address the absence of interactive computerized tools designed to assess the unique needs of children living with OI. Sisom (DHealth; Norwegian acronym derived from *Si det som det er*, meaning *Tell it as it is*) is an award-winning, rigorously tested, interactive, computerized tool that helps children aged 6-12 years with chronic conditions to express their symptoms [22]. It can be downloaded on a phone, tablet, laptop, or desktop computer. Sisom, originally designed for children with cancer, was adapted for children with congenital heart disease, and in the process of being adapted for other conditions, it also has the potential to engage children with other chronic conditions in their own care [23]. It is also considered a creative system that helps clinicians better understand children’s perspectives [22]. Sisom uses spoken text, sound, and animations to depict symptoms that are each represented by an animated scene within 1 of the 5 symptom islands (Figure 1).

After first creating an *Avatar*, one is then prompted by the Sisom Navigator *Mary*, the nurse, to indicate the presence and severity of symptoms displayed by using a 5-point Likert-type scale. Upon completion, Sisom generates a child-friendly symptom report that can be shared with the family and clinicians [22,24].

To date, the development, redesign, validation, and evaluation of Sisom demonstrate the ability of this tool to create a unique environment to elicit children’s perceptions of their condition and engage them in their health care [22,23,25-28].

### Objective

The aim of this study is to describe the experiences shared by children with OI when approached as experts in the co-design of *Sisom OI*, to further advance the prototype development, and to understand the children’s experiences.

## Methods

### Design and Setting

Following the approval of the institutional review board (A06-B29-17B), a secondary analysis of qualitative data was conducted [29]. The data sources consisted of interview transcripts, drawings, field notes, and observations derived from interviewing 12 children with OI who participated in the study aimed to develop the *Sisom OI* paper prototype [13]. Both studies were conducted at a university-affiliated, nonprofit, pediatric, bilingual, orthopedic hospital in Montreal, Québec, specialized in OI care. On the basis of the average prevalence rate of 2 Scandinavian studies [5,6] of 1 in 11,600, we estimated that there were 3190 individuals with OI in Canada, of which the study site has seen 576 children or 18.06% (576/3190) of the population.

### Participants

Purposive sampling was used to allow for maximum variation in age, self-identified gender, and type of OI for children. Children were eligible for the study if they (1) were diagnosed with any type of OI, (2) were aged between 6 and 12 years, (3) received health care at the study site, (4) had a consenting parent or legal guardian, and (5) spoke in either English or French. Sisom was designed with and for children aged 6-12 years to meet their varying cognitive and emotional capabilities [22]. Therefore, the age eligibility criterion was aligned with previous Sisom studies and was limited to this age group. Older children were excluded, as they think and express themselves differently than younger children [22,30]. Moreover, children aged ≥12 years, with enhanced readability, can use other tools [30,31].

The original study sample size estimate of 10-15 children and 5-10 clinicians was proposed based on leading experts [13,32,33]. These sample estimates were also aligned with previous Sisom studies with similar designs to successfully develop the prototype [22,23], translate [25], and establish the usability of the tool [27]. Although other Sisom studies have included larger samples, these studies were quantitative (N=100) [26] or a secondary analysis of 2 studies [25,34], which explicitly increased the sample size to target the multiple disease groups inherent in oncology from multiple sites (n=39) [35]. In this study, a secondary analysis of 12 interview transcripts was deemed sufficient for investigation, as the interviews were rich in children’s verbal expressions of experiences. Finally, data saturation was reached, and a sense of closure was attained because the data derived from our sources yielded redundant information [36].

### Recruitment

The children with OI were recruited by reaching out to clinicians who assisted by identifying, screening, and approaching families to determine if they were interested in hearing more about the study [13]. One member of the study team was charged with providing a verbal and written explanation of the study to those interested in obtaining written parental informed consent and child assent [13].

### Data Collection and Procedures

Data were collected in the context of the participatory co-design of the *Sisom OI* paper prototype [13]. The early stages of the co-design of the *Sisom OI* paper prototype consisted of 3 feedback cycles with 2 to 6 semistructured, face-to-face, individual child interviews per cycle [13]. The length of the interview depended on the child’s interest and varied from 20 to 60 minutes. It was the parent’s or legal guardian’s choice to be present during the interview. During the co-design of *Sisom OI*, the children were prompted in several ways. First, Sisom prompted the children both verbally, through the questions Mary, the nurse, was asking, and visually, through the different animations that appeared. Mary’s questions prompted the children to share their experiences related to each symptom being assessed. Second, the interviewer verbally prompted the children to share and elaborate on their experiences when prompted. The interviewer would either directly ask the child a question or reflect something that the child shared in common with other participants to prompt a response. These prompts helped children to draw from their experiences to determine whether the Sisom symptoms, vignettes, or avatars were relevant or irrelevant, needed to be modified, required new additions, or remained unsure [13]. Third, if present, the parent or legal guardian also prompted their children to share their experiences (Multimedia Appendix 1). Field notes were recorded during and immediately after each audio-recorded interview, which included detailed descriptions of nonverbal data, other observations, impressions, and any drawings generated.

### Data Analysis

The interviews were transcribed and read through, along with the field notes and observations taken by the interviewer and the 6 drawings created by the children [13], to gain a global picture of the data. A descriptive qualitative analysis was then applied to identify themes and patterns identified in the data sources [37]. First, open coding was performed diligently to remain close to the original data. Second, code reduction or clustering was performed to create categories and remove redundant codes [37]. Third, axial coding was performed to find the links between the main categories to generate overarching themes and subthemes. Finally, the original data were revisited to ensure that the child’s experience was properly interpreted and described [37]. Through this process, themes and subthemes across the children’s responses were identified. For each theme and subtheme generated, various examples were used to highlight particular points within the children’s dialogue. Ongoing meetings with the research team were held to discuss how to answer the research question, explore how the data could be best summarized, and check whether designated quotations fit the proposed themes and subthemes. The selected French quotes were subsequently translated into English. Throughout the entire process, an audit trail, composed primarily of methodological and analytical documentation, was kept, permitting the reproducibility and transferability of the process.

## Results

### Sample Characteristics

A total of 12 children participated in this study (Table 1). The participation rate was 92% (12/13). No child withdrew from the study.

**Table 1:** Sample characteristics (n=12).

Characteristics			Values
Age (years), mean (SD; range)			9 (2; 6-12)
**Self-identified gender, n (%)**			
	Boy	7 (58)	
	Girl	5 (42)	
**Nationality, n (%)**			
	Provincial (Québec)	5 (42)	
	National (Canada)	3 (25)	
	International	4 (33)	
**Languages spoken at home, n (%)**			
	English	4 (33)	
	French	4 (33)	
	Bilingual (English and French)	1 (8)	
	Bilingual (English and other)	3 (24)	
**Type of osteogenesis imperfecta, n (%)**			
	I	4 (33)	
	III	2 (17)	
	IV	5 (42)	
	VI	1 (8)	
**Current fracture^a^, n (%)**			
	Yes	5 (42)	
	No	7 (58)	
**Use of mobility devices, n (%)**			
	Wheelchair	2 (17)	
	Wheelchair and walker	4 (33)	
	None	6 (50)	
**Reason for presence at the study site, n (%)**			
	Physiotherapy and occupational therapy	6 (50)	
	Regular checkup	1 (8)	
	Admission	4 (34)	
	Other	1 (8)	

^a^*Current fracture* referred to whether the child was immobilized and recovering from a fracture at the time of interview.

### Child Interview Findings

#### Overview

In the co-design of *Sisom OI*, different prompts allowed children to open up about their experiences of life with OI. These prompts were from Sisom, the interviewer, and, if present, the parent or legal guardian. The dynamic between the child and their parent or legal guardian varied. Sometimes, the parent or legal guardian answered directly for the child. In these cases, the child would then agree, disagree, or remain silent. At other times, the parent or legal guardian gave the child space to answer on their own. The experiences shared in this analysis focused explicitly on the children’s responses. Three themes and their respective subthemes were identified (Multimedia Appendix 2).

#### Relating to Others

**Figure 2:**
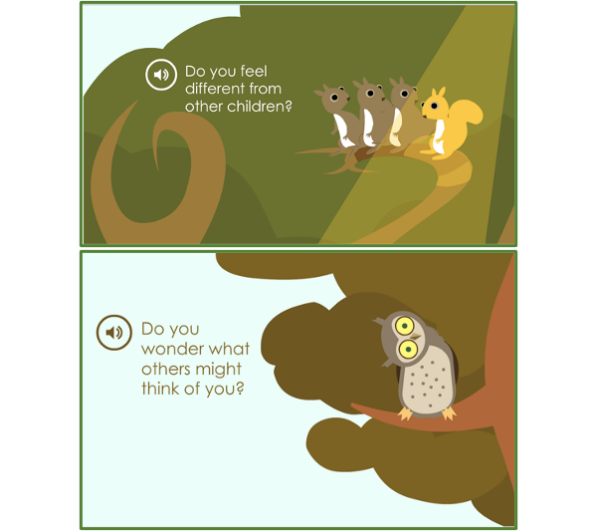
The Sisom graphic that depicts the theme “Relating to Others.”

Children with OI described how they saw themselves in relation to other children. They demonstrated that they were different but also very similar to any other child (Figure 2).

##### Common OI Experience

Children with OI have a distinctive experience that unites them. They all recognize their integral role within the OI community. Children used terms such as “OI kids” [P5, 10 years old, type IV OI], “We” [P5, 10 years, type IV OI], and “Us” [P7, 9 years old, type III OI], showcasing this common OI experience. One child with an extensive OI history used her experience to speak on behalf of other members of the OI community stating “Well, OI kids, we as, since I’m an OI kid, [...] since I know...” [P5, 10 years old, type IV OI]. Some children seemed to adopt advocacy roles. One child described how families with children with OI look out for one another. He explained how his mother developed a *break* kit for other OI families:

We had a friend that lived 45 minutes away from us that had OI and he broke and he has new parents, like he’s one year old, so they don’t know how to splint. So mum went over and splinted him so that’s how we thought of it!

P12, 10 years old, type IV OI

One child answered his questions about Sisom by thinking of others:

I think, we have to like, keep this because, some people, it’s hard [...]...Some people are impatient.

P4, 8 years old, type IV OI

Another child put herself in others’ shoes and remarked that older children have more difficulty than younger children in asking for help:

It’s possible, when you’re younger, that it doesn’t bother you, but when you’re older, it bothers you more. It embarrasses you. You want to do everything yourself...So there’s a big difference.

P11, 11 years old, type IV OI

One child advocated for the creation of *Sisom OI* in other languages so that it could be used by more children (P12, 10 years old, type IV OI). All children showed profound empathy for others with OI in their responses. Those who were more novice asked questions to the interviewer to understand the experience of others to answer Sisom’s prompts: “Probably no problem, but I never tried it though, by the way” [P6, 6 years old, type I OI]. Those who were more expert related their own experience to how others might feel:

Sometimes people are, are so like, you don’t know what you want, but that happens to me sometimes and sometimes people will cry because of that. Like me.

P4, 8 years old, type IV OI

##### Feeling Different

Children were asked by Sisom if they felt different than other children (Figure 2). They all responded that they did. One of them stated matter-of-factly: “Ya...because other kids are different than us.” [P5, 10 years, type IV OI]. They also expressed that feeling different affected their lives in several ways. First, many of the children reflected that their differences led to being bullied. One child expressed how her peers at school teased her because she was different: “They often say, let’s say, that I don’t run fast, and they laugh at me.” [P8, 7 years, type I OI].

Second, they reflected that their differences led others to stare at them, “the gaze of others” [P3, 11 years, type IV OI], which they acutely sensed, and caused their worries about feeling different to amplify. One child described how people gawked at her when she went out to the restaurant: “It’s that I often get stared at, at the restaurant, and I find that unpleasant” [P3, 11 years old, type IV OI]. Other children described strangers directly asking them questions about their bodies and having to explain their condition to others. For some, this was frustrating. In contrast, 1 participant did not let these types of questions hold her back from sharing knowledge with others about her condition:

I just tell them to “Be careful! Because I could get hurt more easily than you.” I remember that there was someone that told me at Burger King: “Why are you more fragile than others?” I just explained to him that “Well, I have an illness, so my bones are more fragile and that if I fall it could break me...” and he was like “Ah...” Because [...] he kind of bumped into me.

P11, 11 years old, type IV OI

Third, they reflected that their differences led others to treat them differently. One child described how others were afraid to break her bones and that she was ostracized on a daily basis:

I feel very lonely. No one wants to play with me because of my illness.

P8, 7 years old, type I OI

Other children expressed that people’s perceptions of the hardships of living with OI were overexaggerated. One child recounted the story of a stranger who wanted to give her a hug because she felt pity for her [P3, 11 years old, type IV OI]. Another child reflected on how he felt when his mother was overprotective of him:

That’s annoying! I hate that. Be careful! You might get hurt.

P7, 9 years old, type III OI

However, 2 children viewed being different as positive, stating:

Everybody’s different

P4, 8 years old, type IV OI

Being different from other kids is a good thing. Like, for example, I think that because I’m in a wheelchair, I can do things that other kids can’t do, and that, that part’s a good thing.

P5, 10 years old, type IV OI

##### Feeling Just Like Others

All children demonstrated that they were more similar to others than they were different. The children shared their desires, wishes, and preferences just as any other child would. The goal was to engage children in the co-design of the *Sisom OI* paper prototype; however, OI was sometimes left out of the conversation altogether by the participants. For instance, when 1 child was asked what makes them feel different from other children, they commented on their age. When asked if there was anything else, they stated, “That’s it” [P10, 9 years old, type VI OI]. Every interview included children laughing, playing, and sharing, just as any other child would.

They shared things about themselves that they found special, such as their likes: “We all love unicorns!” [P6, 6 years old, type I OI]; their dreams: “I want to be a vet.” [P5, 10 years old, type IV OI]; and their hobbies:

I am a HUGE Harry Potter fan! I am at the 5th book.

P7, 9 years old, type III OI

One child, by having people get to know her, showed others that she was more than just an *OI kid*:

They just don’t know me yet. So like, [...] if a friend was there, then they wouldn’t look at me like that, cause they’d know that it’s my, I have a condition...

P5, 10 years old, type IV OI

This child suggested the creation of a Sisom island called *About Me* in which children could share what was unique about them. This child also emphasized that the avatar they create of themselves within *Sisom OI* be as realistic as possible. This view was shared by all participants: “You would like [...] that it looks the most like you...” [P7, 9 years old, type III OI] so that “You could see yourself...Through a computer technological program” [P4, 8 years old, type IV OI]. Many of the children had fun creating their avatars and customizing them:

Ya, I like the color on that one!

P9, 6 years old, type III OI

I’d like to put some shoes I like to wear. I got light up shoes but I’m not wearing them now...

P10, 9 years old, type VI OI

Many of the children reflected how they were the same as everyone else by normalizing their symptoms, stating:

Everyone’s gonna feel like that, right?

P7, 9 years old, type III OI

It’s pretty much [like that for] everyone.

P3, 11 years old, type IV OI

They often described how they felt the same way others felt and shared many of the same experiences. Similarly, when 1 child was asked about her nightmares, she answered:

I have more nightmares about like clowns and animals and stuff...That’s what scares me. Not really surgeries.

P11, 11 years old, type IV OI

The same participant explained how she adapts her situation to be able to participate in events just like any other child. For instance, she takes her wheelchair out to go “trick-or-treating” to make the most of it:

For example, Halloween, I would never do that with my walker! I would never be able! [...] By the time I get there wouldn’t be any candy left!

P11, 11 years old, type IV OI

#### Relating to Their Condition

##### Overview

**Figure 3:**
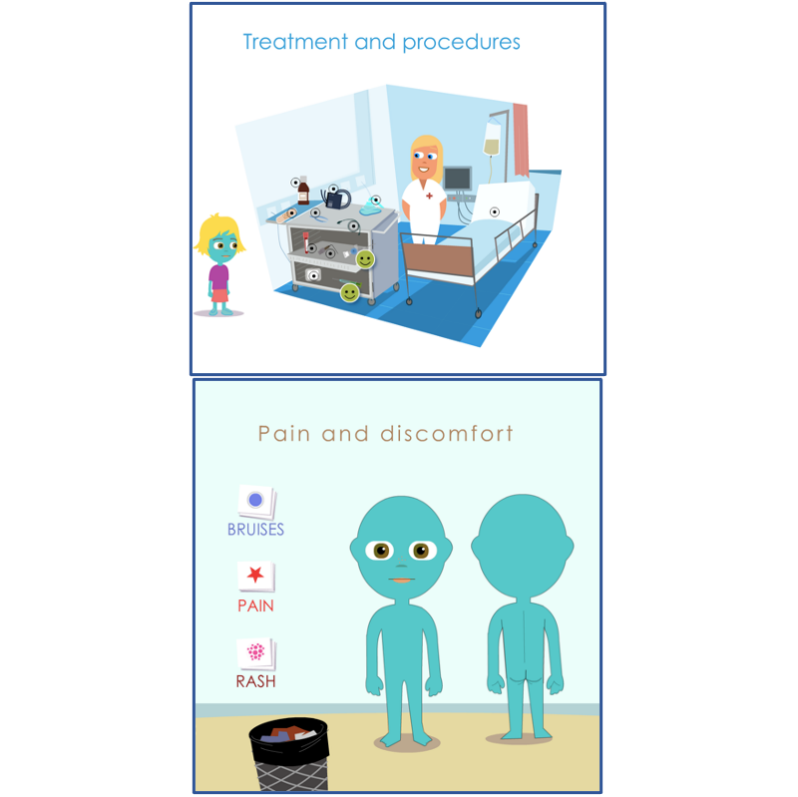
The Sisom graphics that depict the theme “Relating to Their Condition.”

In *Relating to Their Condition*, children described their positive and negative interactions with their own condition and the health care system (Figure 3).

An example of a child’s quote on *Relating to Their Condition* is as follows:

The problem with OI is not how you look, it’s like, how you feel...is there any pain? Stuff like that! Not how you look! This is actually...That’s what makes sense to me.

P4, 8 years old, type IV OI

##### Understanding Their Condition

Each child interviewed had a thorough understanding of their OI. The depth of their knowledge seemed to correlate with the breadth of their experiences within the health care system. Children who were older with more severe OI had more extensive experiences with treatments, procedures, and the hospital overall. Within our sample, children ranged from knowing the general principles and basics of OI to being very informed and involved in their own health.

Some children only had rudimentary knowledge. One child was asked about her experience with OI. She simply replied:

No, I don’t know that [OI] [...] I come here to get my bones looked at.

P6, 6 years old, type I OI

Similarly, another child, when asked about OI, stated:

What is that?...I have brittle bones like I can just hit [...] something and [...] get hurt.

P10, 9 years old, type VI OI

Most children understood that their condition was a hereditary genetic disorder that affected their bones and caused pain. One child described it as, “Having pain in my bones” and “For example, I could have had brothers and little sisters with this disease” [P8, 7 years old, type I OI].

In contrast, one child knew the names of many of his bones, medications, and their indications as well as the pathophysiology of OI:

This arm, before it was operated, it was like a banana. [...] And it was because, my muscles, they’re fine. But, it’s that, they pulled on my bone, since my bone is like, so much like rubber, it like, weep [sound].

P7, 9 years old, type III OI

One child even described her disease in a metaphor to be able to help others understand:

Because, the OIs, they say “bones of glass.” So maybe [we could show] a glass that is broken.

P11, 11 years old, type IV OI

The way in which these children interpreted and related their experiences with OI to others seemed to depend on their level of involvement with their own health. However, each child chose a similar way of expressing what it meant to have OI to them by physically showing where they had pain (P6, 6 years old, type I OI), discomfort (P3, 11 years old, type IV of OI), and surgical scars (P7, 9 years old, type III OI). One child got up on a chair to show from what height she might need to fall to break a bone (P6, 6 years old, type I OI). Another child, when approached about how he felt about his appearance, stated:

The problem with OI is not how you look, it’s like, how you feel...is there any pain? Stuff like that! Not how you look! This is actually...That’s what makes sense to me.

P4, 8 years old, type IV OI

##### Special Relationship With the Hospital

Children with OI reported spending significant amounts of time in the hospital. As put positively by 1 child, “Sometimes I say the hospital and I are friends cause OI kids, we go to hospital a lot, for check-ups and stuff” [P5, 10 years old, type IV OI]. Children described many long and boring days in the hospital. One child expressed:

...Some patients with surgeries, they like...I don’t know they like, get bored, in the, sitting in the room in a bed all day long.

P4, 8 years old, type IV OI

Another child described the dread of staying overnight:

Mostly I am scared of having pain when I turn,...I am scared of spending a bad night after my surgery [...] It’s cause you never know [...] if you’ll sleep, if you won’t sleep...

P11, 11 years old, type IV OI

Children also missed their home, family, and friends. One child expressed not liking her treatments at the hospital because of “The fact that I’m far away from my sisters” [P8, 7 years old, type I OI]. Another child described missing home during a long stay after her spinal surgery:

We stayed there for a like a long time. Like one week, and I got sooo homesick. I missed like the city, the hustle and bustle, I missed my friends.

P5, 10 years old, type IV OI

It was also common for children to travel from great distances to receive treatment, as it was for one child, whose mother had to stay home, in another province:

Whenever I call mom I start to cry [...] She can’t come here cause she has to work.

P6, 6 years old, type I OI

The hospital became somewhat of a home for many of these children who had formed a special relationship with the space and staff. For example, 1 child described his experience in the playroom:

Back when we had surgery, here, we always loved to go to the playroom [...] The volunteers, I actually like the volunteers, like I made a friend [and...] we made a made-up movie together.

P4, 8 years old, type IV OI

This special relationship also extended to the physicians, in whom the children placed a lot of trust to help them get better. One child described her fear of dying during surgery and that she placed trust in her physicians to help her through:

I trust doctors...cause they want to help me feel better. [...] Surgeons, yeah.

P5, 10 years old, type IV OI

##### Difficult Treatments and Procedures

Children described having to undergo countless unpleasant and painful procedures. When asked about medications, almost all the children expressed that they tasted horrible:

*Blerrrk!* [P8, 7 years old, type I OI]

...sometimes when I eat medicine, my taste buds are screaming at me like “WHY ARE YOU EATING THIS?”

P5, 10 years old, type IV OI

Another common and dreadful experience was having to undergo surgery. One child enumerated 3 different reasons for requiring surgery because of OI, including coxa vara repair, scoliosis repair, and rerodding. She described her experience with surgeries:

I have a lot of surgeries, when you have surgeries and you fall asleep for the first time, it’s like very difficult, cause I see a lot of nightmares.

P5, 10 years old, type IV OI

It was something that she had a lot of experience with and to which she was now getting accustomed. This was similar to another child who showed off the many scars from a number of different surgeries [P7, 9 years old, type IV OI]. Another child emphasized how often he had surgeries:

Sometimes like, if I get hurt and I hear my leg crack [...]. They go in, and then, if I didn’t have a rod in, they put a rod in, and that’s how they do it...I did this MANY times.

P10, 9 years old, type VI OI

Children not only expressed that they felt discomfort during hospital procedures but also shared their pride in having gotten used to certain painful procedures. One child described being “Brave for everything” and doing a “Good job” by not crying during painful procedures, which she described as “another unhappy task” [P6, 6 years old, type I OI]. Another child justified his painful experiences with their purpose:

IVs

intravenous accesses] are, they’re good. But like, so like you could have medicine...But, then again IVs do hurt a bit. But, they’re good so...[P4, 8 years old, type IV OI

In addition, the children reflected that tolerating pain came with experience. One child reflected on how he had come so far:

Not a problem, for shots, for me...It’s been a long...At the beginning I couldn’t...

P7, 9 years old, type III OI

#### Reflecting on Capabilities

##### Overview

**Figure 4:**
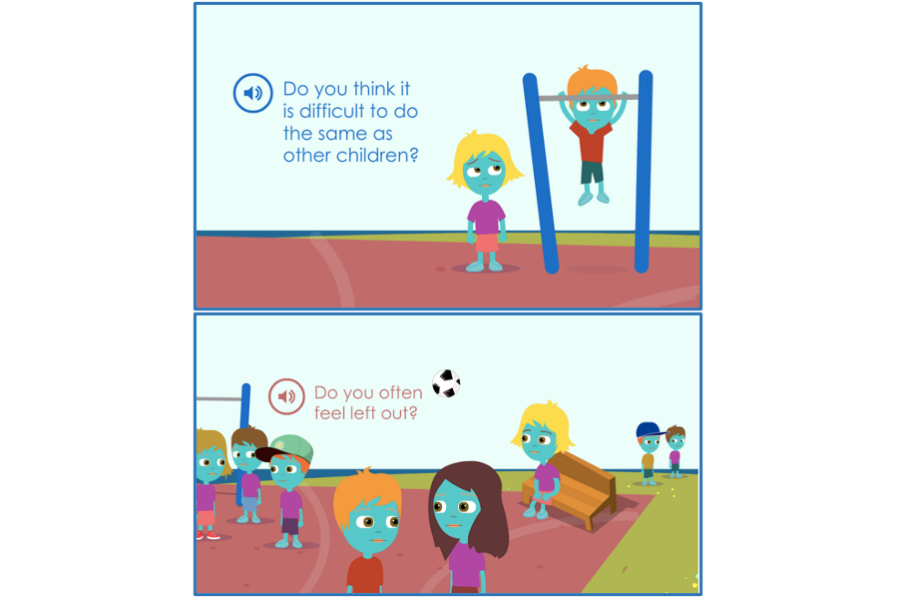
The Sisom graphics that depict the theme “Reflecting on Capabilities” and the subtheme “Overcoming Isolation.”

By reflecting on their capabilities, children were able to identify some of their strengths and weaknesses (Figure 4).

An example of a child’s quote on *Overcoming Isolation* is as follows:

I do not like playing outside [alone] because everybody excludes me. [...] I like playing outside even though I have this condition, because I have many friends at school.

P7, 9 years old, type III OI

##### Perceiving Limitations

All children expressed a desire to do something they were physically incapable of because of a fear of getting hurt. For some children, this activity was eating. Some described not being able to eat tasty foods that were “Like, let’s say, sticky things...and things that are too hard” [P8, 7 years old, type I OI] and “like [...] bars, for example, I can no longer have them...” [P3, 11 years, type IV OI] because of an orthodontic mouthpiece they were required to wear for their OI. One child explained that OI prevented her from playing with her friends:

Like for example...there, like, in my school yard, there’s like a big slide [...] where you climb up high, it’s like twisty. [...] so whenever I see them, my friends, they’re going down the slide, they’re having so much fun, it’s like, I can’t do it.

P5, 10 years old, type IV OI

Another explained that his OI led to fractures that required him to wear a cast for healing. During this period, he was not able to play sports with his friends [P7, 9 years old, type III OI]. This sentiment was echoed by another child who drew a picture to show how it felt to be excluded because of an injury:

Like, if he’s looking at his cast, and his friends are outside playing street hockey...He’s in his room [...] It affects you to watch your friends play and you have to stay inside doing nothing.

P11, 11 years old, type IV OI

Children also expressed how their perceived limitations may affect their future endeavors. One child expressed her desire to be a good dancer but that this was difficult because of the way her OI affected her bones. She reflected:

Well, cause, I...I just wanna be a good dancer, and like, I wanna have strong bones...[P6, 6 years old, type I OI]

Another child stated that it would be important to include the question “Are you afraid that your handicap will prevent you from doing what you really want in life?” [P11, 11 years old, type IV OI] in *Sisom OI*. She explained how a person with OI may want to become a police officer but would have to settle for working in the office answering phones instead of being out in the field.

Some children also reflected on what their lives might have been like without OI. One recounted her daydreams and how she would “Go to any place [...] hang out with [her] friends alone, [...] help [her] mom a lot, [...and] go around [...] to places with [her] dad” [P5, 10 years old, type IV OI]. She reflected on needing help for some activities for which she was dependent on others. She also asserted her independence for other tasks. Most of the other children reported a similar balance.

##### Overcoming Isolation

Ideas of feeling left out were expressed by all children. When prompted by Sisom “Do you ever feel left out?” children answered:

A lot, because, because no one, almost no one, wants to be with me because of my disease.

P8, 7 years old, type I OI

Sometimes, [...] cause sometimes kids, they don’t wanna play with you, or, because of your condition they don’t wanna talk to you...

P5, 10 years old, type IV OI

They overcame these feelings by finding independent activities and excelling in other areas, escaping through imagination, having good friends, and thinking positively.

First, the children asserted their independence in several areas such as reading and journaling and taking time for themselves to be alone: “I want to write something on here!” [P9, 6 years old, type III OI]. One child described how she turned to nature when her sister did not want to play with her: “So I just go outside and sit behind one of my trees” [P6, 6 years old, type I OI]. Another child described how while she was dependent on others in some areas, she was determined to find ways to maintain her independence:

When I’m at school, I have an aid, but she doesn’t go in the bathroom with me, she just holds the door in case I fall [...] I do my things, and I leave. She doesn’t come in with me, I do it all by myself.

P11, 11 years old, type IV OI

Second, building and maintaining strong friendships helped children overcome the feeling of being left out. One child referred to her pets when she reflected that “at least I have friends” [P8, 7 years old, type I OI]. Another explained that good friends made feelings of exclusion tolerable:

I do not like playing outside [alone] because everybody excludes me. [...] I like playing outside even though I have this condition, because I have many friends at school.

P7, 9 years old, type III OI

One child reflected how she did not feel bad when her friends played without her at recess but always appreciated the time that they spent with her:

They climb the spider webs, and all those things, and me, I couldn’t do them, so I stayed sitting at the table with my friends and we talked...Sometimes they left and they came back...To have fun a bit [...] I wasn’t going to prevent them from doing everything and say: “No you stay with me because I can’t do it” [Laughs].

P11, 11 years old, type IV OI

Third, they used their imagination to escape to different worlds by making up “a game like BOOM” [P7, 9 years old, type III OI], having an “imaginary friend” [P7, 9 years old, type III OI], imagining a “candy world, [...] a land full of candy and unicorns” [P6, 6 years old, type I OI], and “daydream[ing] about [the] future [...] working with animals” [P5, 10 years old, type IV OI]. Positive thinking was implicit in the attitudes of all children. One child embraced this philosophy when she explicitly stated:

As a person, you always have to think positive. Cause if you think about the negative, then you’ll see life in a bad way. But life is not as bad as you think.

P5, 10 years old, type IV OI

##### Celebrating Strengths

All the children experienced feelings of otherness, exclusion, and physical limitations, but they also celebrated their strengths and asserted their independence in various ways. Children were excited about their mobility improvements:

I am quickly learning how to walk.

P8, 7 years old, type I OI

[running], that’s the next step.

P7, 9 years old, type III OI

Some expressed confidence and pride in their current mobility status:

I’m an expert driver with my wheelchair!

P4, 8 years old, type IV OI

You know, I go really fast in my wheelchair.

P3, 11 years old, type IV OI

This confidence allowed them to attend special events and show off their skills. One child participated in special Paralympic tournaments: “I won [...] I got three gold medals” [P3, 11 years, type IV OI]. She also played soccer at her specialized school, where she felt comfortable being herself.

Another child also played soccer and hockey with his friends [P7, 9 years old, type III OI]. These adapted environments were important contributors in reinforcing feelings of inclusion and value.

The children also celebrated their academic accomplishments. They highlighted how well they do in school: “I’m one of the best readers in my class!” [P12, 10 years old, type IV OI]. The children described their accomplishments with pride and confidence. One child explained how she was able to excel even in an accelerated program:

I am in the intensive stream [...] So I do my sixth grade in six months...[Actually] five months! [...] So I have to go quickly! It’s like I’ve become a model!

P11, 11 years old, type IV OI

Finally, children with OI seemed to truly look out for one another. Their involvement in this study reflected kinship. Many participants understood the research implications for other children with OI. They highlighted the importance of including “everybody’s ideas, everybody’s advice” [P7, 9 years old, type III OI] and hoped:

that kids will love it! Cause, we need to express it in a way that the public will love it [...] So we can make money and we can help this disease!

P4, 8 years old, type IV OI

This kinship was further exemplified by one child who was very implicated in fundraising for OI. She made it a point to encourage other children with OI to keep their heads high:

From my point of view, OI kids, they don’t think that they’re [...] awesome. [...] But you are awesome. I sometimes tell OI kids that, cause like, most of the ones that I know, they’re like young, like five or six, [...] So I tell them that: “You are awesome, cause you went through so much, and you’re so little!”

P5, 10 years old, type IV OI

Not only did she celebrate her own strengths, but she also encouraged others to find their strengths and celebrate them.

## Discussion

### Principal Findings

Efforts to *authentically listen* and bring to light what children with OI shared when approached as experts in their condition was made possible by conducting a secondary analysis of qualitative data sources collected for the co-design of the *Sisom OI* paper prototype [13]. This study demonstrates, as shown in other recent findings, that children who use Sisom are prompted to share their experiences [25,27]. Through the analysis of their dialogue, 3 themes were identified. The first, *Relating to Others*, described the balance between feeling different versus feeling similar to other children. The subthemes were *Common OI Experience*, *Feeling Different*, and *Feeling Just Like Others*. The second, *Relating to Their Condition*, described children’s positive and negative interactions with their own condition and the health care system. The subthemes were *Understanding Their Condition*, *Special Relationship with the Hospital*, and *Difficult Treatments and Procedures.* The third, *Reflecting on Capabilities*, described children’s recognition of their strengths and limitations. The subthemes were *Perceiving Limitations*, *Overcoming Isolation*, and *Celebrating Strengths*.

Children with OI had a unique way of maintaining balance in their lives. This was demonstrated through all three of the themes. First, in *Relating to Others*, children described a balance between feeling different and feeling similar to other children. They also expressed a concern about the perception of others. This has previously been described in other studies of children with OI [8,38]. An important way that children with OI overcame this feeling of otherness was through building relationships with other children and clinicians in the OI community [8,39]. Another way children navigated between feeling different and feeling similar to other children was by sharing with others the special things that defined them and by participating in activities with other children as best as they could. This helpful strategy has been described in other populations of children with chronic conditions [17,40].

Second, in *Relating to Their Condition*, children described a balance between the positive and negative aspects of living with OI. Children’s depth of understanding and moral awareness can range from *rich sophistication* to *simplistic matter-of-factness* [20]. Children are capable of such awareness, and their voice should be attended to without comparison with the adult perspective [20]. Indeed, in the data, there was a wide range of depth in the understanding of OI. All children were able to share what it meant to have OI. Being in the hospital was an experience that was painful, with numerous unpleasant procedures including surgeries, needle pricks, and extended hospitalizations. The fear of needles was encountered by children living with OI in this study as well as others [8,38]. Tolerating pain and *getting used to* painful procedures was something that children with OI have expressed in other studies [38]. This idea of *getting used to* pain was expressed by the children as a proud adaptation to a difficult aspect of living with OI. Another way that children overcame the negative aspects of being hospitalized was by reflecting on the positive relationships they had developed with clinicians and volunteers, which other children have reported [18,41]. It has been shown previously that children with OI form special relationships with people who understand OI, for instance, their physicians [8].

Finally, in *Reflecting on Capabilities*, children described a balance between perceiving their limitations and celebrating their strengths. Feeling left out because of the fear of fractures or physical limitations was a major element of this theme and has been explored in other studies [8,38,42]. Despite the presence of this feeling, children with OI have been found to have an immensely positive and strength-based outlook on life [8,39,42]. Our study showed 1 way that these children embraced positivity was to constantly strive to improve themselves physically and academically. This concept of a strong desire to move forward was supported by other studies; however, these studies also showed that children with OI held back due to the fear of pushing too hard and fracturing [8,39]. Children’s perception of these physical limitations propelled them to take on and excel in their intellectual feats [8]. Interestingly, our study showed that, given the right environment and support, children with OI are also capable of excelling in physical feats. These children are *taking life in stride* and using their positive outlook to overcome feeling different and feeling left out and making the best of every day [42].

Sisom may contribute to transforming a child’s *place* into a child’s *space* [13] by inviting children to participate in their care [28] and showcasing how they can serve as advisors and partners in their care [21]. Sisom allows for the promotion of discussions about what children deem the most important. This essential information may help clinicians enhance children’s participation in their own care and ultimately empower them to cope with the difficult physical, mental, and social challenges they face. However, *Sisom OI* is yet to be developed, tested, or integrated into practice. Therefore, these study findings offer only preliminary insight into the types of discussions that may unfold from the future use of *Sisom OI* in practice. As OI remains a rare disease with few clinicians encountering a child with OI over the course of their career, there may be some reluctance to use Sisom. Offering training and delineating potential responses to children’s expressed concerns may help integrate this novel, interactive, computerized tool into practice and contribute to the transformation of children’s rightful space in their health care systems [28,35].

In this study, the partnership created between the interviewer and the child successfully elicited and highlighted the *child’s perspective*, placing the child in the center of their experience. These children had many things to share about their experience, as noted by other chronically ill children [43]. Although parental involvement, at times, made it difficult for the child to take the lead, our approach revealed the child’s perspective. Parents highlighted the things with which children did not necessarily agree. These differences in perspective have been reported in previous studies [15,16]. Sisom’s ability to highlight such differences is considered a key feature, as it strives to promote communication between children and adults so that they may engage in shared decision-making.

### Limitations

The children in this study were prompted to give their opinion on Sisom to co-design *Sisom OI*. Therefore, they were not directly prompted to share their OI experiences, unless prompted by the interviewer. The questions posed in Sisom were not tailored to specifically capture the experiences of children with OI. This may have led to significant data gaps. Future studies should be conducted to specifically aim to understand children’s perspective on their OI.

Furthermore, the involvement of parents and legal guardians in the interviews had, in some cases, a muting effect on the child. Instead of giving space for their child to answer the questions independently, some took it upon themselves to answer for their child. In some cases, this prompted the child to express their feelings and share their experiences, whereas in other cases, this caused the child’s voice to be lost. Further studies should consider how to highlight the child’s voice by emphasizing on the child’s answers and constantly reemphasizing the purpose of the study to the parents involved.

### Clinical Implications

This study highlights the richness of the experiences shared by children who are approached as experts in their own condition. These children had valuable information and insights to share regarding their day-to-day lives and the factors in their lives that both hinder their development and support their endeavors. The study findings offered a glimpse of what it means to grow up living with OI but also showcased a novel approach to gain their perspective. The children’s inputs will be further used to advance the development of *Sisom OI* and our overall approaches to offer care that responds directly to their needs and empower them to be active partners in their care.

### Research Implications

The study findings will be also used to inform the development, verification, validation, and evaluation of *Sisom OI* to ensure that meaningful aspects of life with OI are included in the app and attended to in practice. At present, the *Sisom OI* paper prototype has been developed [13]. Although many of the existing Sisom symptoms were deemed relevant for inclusion in the *Sisom OI* paper prototype, 57 new symptoms were generated. The relevant symptoms addressed children’s thoughts and feelings about hospitalization and their wishes for participation in their own care. The new symptoms addressed fractures, body image, and social isolation related to difficulties with accessibility and intimidation. Therefore, these findings, in addition to the themes identified in this study, will be used to inform the next phase of *Sisom OI* development with end user partnerships, stakeholder buy-in, and the necessary funding. We expect to build the prototype; test for usability; pilot for feasibility; and evaluate the integration of *Sisom OI* into the hospital, home, and school settings.

### Conclusions

This co-design process provided children with OI the space to not only contribute to the development of the end product but also to eloquently describe their experiences. These findings, based on the description given by the children themselves, offer us a unique understanding of what it means to grow up with OI.

## Appendix

Multimedia Appendix 1 Child interview guide.

Multimedia Appendix 2 Themes and subthemes identified when children were approached as experts in the co-design of Sisom osteogenesis imperfecta.

## Abbreviations

OI: osteogenesis imperfecta
