# Supplementary material for: Experiences of Children With Osteogenesis Imperfecta in the Co-design of the Interactive Assessment and Communication Tool Sisom OI: Secondary Analysis of Qualitative Design Sessions
Source: JMIR Pediatr Parent. 2021 Aug 10;4(3):e22784. doi: 10.2196/22784 (PMC8386389; doi:10.2196/22784)
Supplement: Multimedia Appendix 1 [file pediatrics_v4i3e22784_app1.docx]

**Multimedia Appendix 1 – Child Interview Guide**

**Interviewer Procedures**

Prior to turning on the audio recorder review signed consent and assent forms with child and parent(s) or legal guardian(s). Ensure voluntary nature of the interview process. Ensure it is understood that all information shared will remain confidential. Turn on the audio-recorder. Permit the child to log on to Sisom. Permit the child to create an Avatar and visit each of the five islands and sub-islands while you ask about relevance. For each element of the Avatar, check off the child’s response: (a) Relevant; (b) Irrelevant; (c) To modify; (d) To add; (c) Unsure. Specify any modifications to elements suggested as well as rationale for any alternative elements proposed. For each symptom, check off the child’s response: (a) Relevant; (b) Irrelevant; (c) To modify; (d) To add; (c) Unsure. Specify any modifications to symptoms suggested as well as rationale for any alternative symptoms proposed. Monitor whether the child or parent(s) or legal guardian(s) had any difficulty in understanding Sisom and track any issues that arose.

**Child Instructions**

Thank you for taking the time to talk to me about Sisom. Sisom is a computer program that helps children to tell about how they are feeling. For our interview, I would like you to visit the Sisom islands. For each island, I will ask you some questions. There are no right or wrong answers. If you do not understand anything, please let me know. During the interview, we can stop at any time you wish, even if we do not finish our interview or go through all the pictures. We can also stop to take a break. I would like to hear what you [name of child] think, so I will ask you my questions first. I am most interested in your words but if [name of parent(s) or legal guardian(s)] thinks they can help you answer, we can ask them for help. I would like to record our conversation, so I do not forget anything you tell me. Before we start do you or [name of parent(s) or legal guardian(s)] have any questions? *(Address questions)*

**Child Interview**

1. (*To be used as an “ice-breaker”)* First, I want to know a bit about you.

a) Where is your favourite place in the world?

b) What topic do you like best in school?

2. Now, I want to show you Sisom. *(Show Avatar)* Here is where you pick what you look like. *(Show choices)* What do you think of these choices?

a) What would you add?

Probe: A walker?

Probe: A wheelchair?

(N.B. Probes will evolve based on previous Child Interviews and suggestions made by each Expert Panel Meeting).

b) What would you take away?

c) What would you change?

Non-Directive Probes

Can you show me what you are telling me?

Can you draw what you are telling me?

Can you say a bit more about that?

Can you say why you think that is important?

Is there anything else you would like to say?

3. (*Show 1 island)* Here is an island. (*Show associated symptoms)* Here are all the problems on this island.

a) What problems are missing?

Probe: “Feel scared of breaking a bone?”

Probe: “Feel left out of activities?”

b) What problems would you take away?

c) What would you change?

Non-Directive Probes

Can you show me what you are telling me?

Can you draw what you are telling me?

Can you say a bit more about that?

Can you say why you think that is important?

Is there anything else you would like to say?

4. (*Show next island(s)).* Here is another island. (*The number of islands shown will depend on the child’s interest.)* Here are all the problems on this island. *(The number of associated problems shown will also depend on the child’s interest.)*

a) What problems are missing?

Probe: “Feel scared of breaking a bone?”

Probe: “Feel left out of activities?”

b) What problems would you take away?

c) What would you change?

Non-Directive Probes

Can you show me what you are telling me?

Can you draw what you are telling me?

Can you say a bit more about that?

Can you say why you think that is important?

Is there anything else you would like to say?

5. *(Repeat “4.” for as many islands as the child is interested in visiting and as many associated problems as the child is interested in seeing.)*

a) What problems are missing?

Probe: “Feel scared of breaking a bone?”

Probe: “Feel left out of activities?”

b) What problems would you take away?

c) What would you change?

Non-Directive Probes

Can you show me what you are telling me?

Can you draw what you are telling me?

Can you say a bit more about that?

Can you say why you think that is important?

Is there anything else you would like to say?

6. (*Show islands)* Now that you have had a chance to see a few islands, let’s take a look at all the islands.

a) What did you think of travelling from one island to the next?

b) What islands are missing?

c) What islands would you take away?

d) What would you change?

Non-Directive Probes

Can you show me what you are telling me?

Can you draw what you are telling me?

Can you say a bit more about that?

Can you say why you think that is important?

Is there anything else you would like to say?

7. (*General report*). What do you think about Sisom?

Non-Directive Probes

Can you show me what you are telling me?

Can you draw what you are telling me?

Can you say a bit more about that?

Can you say why you think that is important?

Is there anything else you would like to say?

8. (*General report*). Would you use Sisom?

Non-Directive Probes

Can you show me what you are telling me?

Can you draw what you are telling me?

Can you say a bit more about that?

Can you say why you think that is important?

Is there anything else you would like to say?

9. (*Overall impression*) What do you like about Sisom?

Non-Directive Probes

Can you show me what you are telling me?

Can you draw what you are telling me?

Can you say a bit more about that?

Can you say why you think that is important?

Is there anything else you would like to say?

10. (*Overall impression*) What do you not like about Sisom?

Non-Directive Probes

Can you show me what you are telling me?

Can you draw what you are telling me?

Can you say a bit more about that?

Can you say why you think that is important?

Is there anything else you would like to say?

**Closing Remarks**

Thank you very much for your help. Here is your Certificate of Participation.

*(Provide ‘Certificate of Participation’)*

Do you or [name of parent(s) or legal guardian(s)] have any questions?

*(Address questions)*
