# Supplementary material for: Experiences of Children With Osteogenesis Imperfecta in the Co-design of the Interactive Assessment and Communication Tool Sisom OI: Secondary Analysis of Qualitative Design Sessions
Source: JMIR Pediatr Parent. 2021 Aug 10;4(3):e22784. doi: 10.2196/22784 (PMC8386389; doi:10.2196/22784)
Supplement: Multimedia Appendix 2 [file pediatrics_v4i3e22784_app2.docx]

**Multimedia Appendix 2 – Themes and Subthemes Identified When**

**Children Approached as Experts in the Co-design of Sisom OI**

| Icons | Themes | Subthemes |
| --- | --- | --- |
| 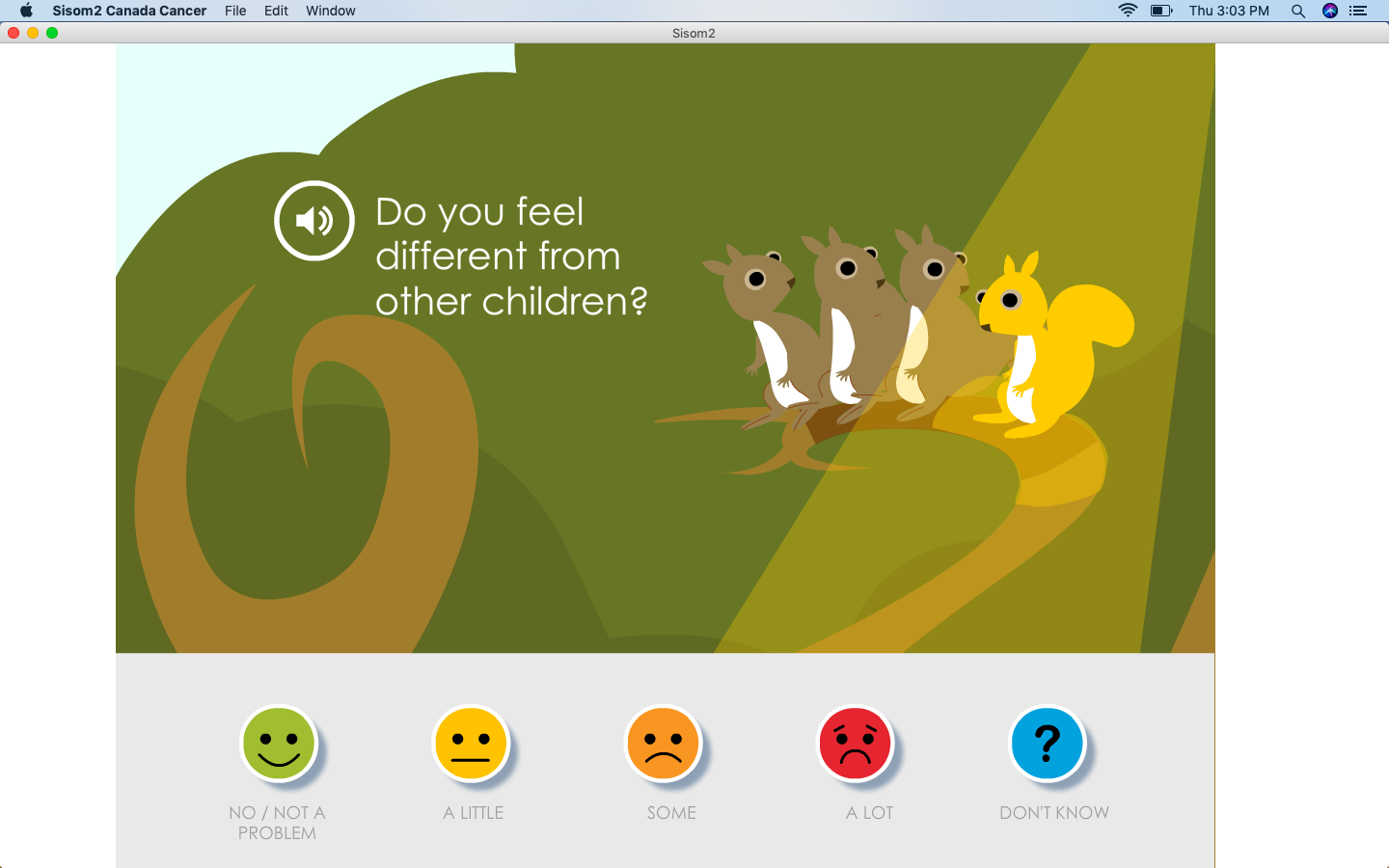 | Relating to Others | Common OI^a^ Experience |
|  |  | Feeling Different |
|  |  | Feeling Just Like Others |
| 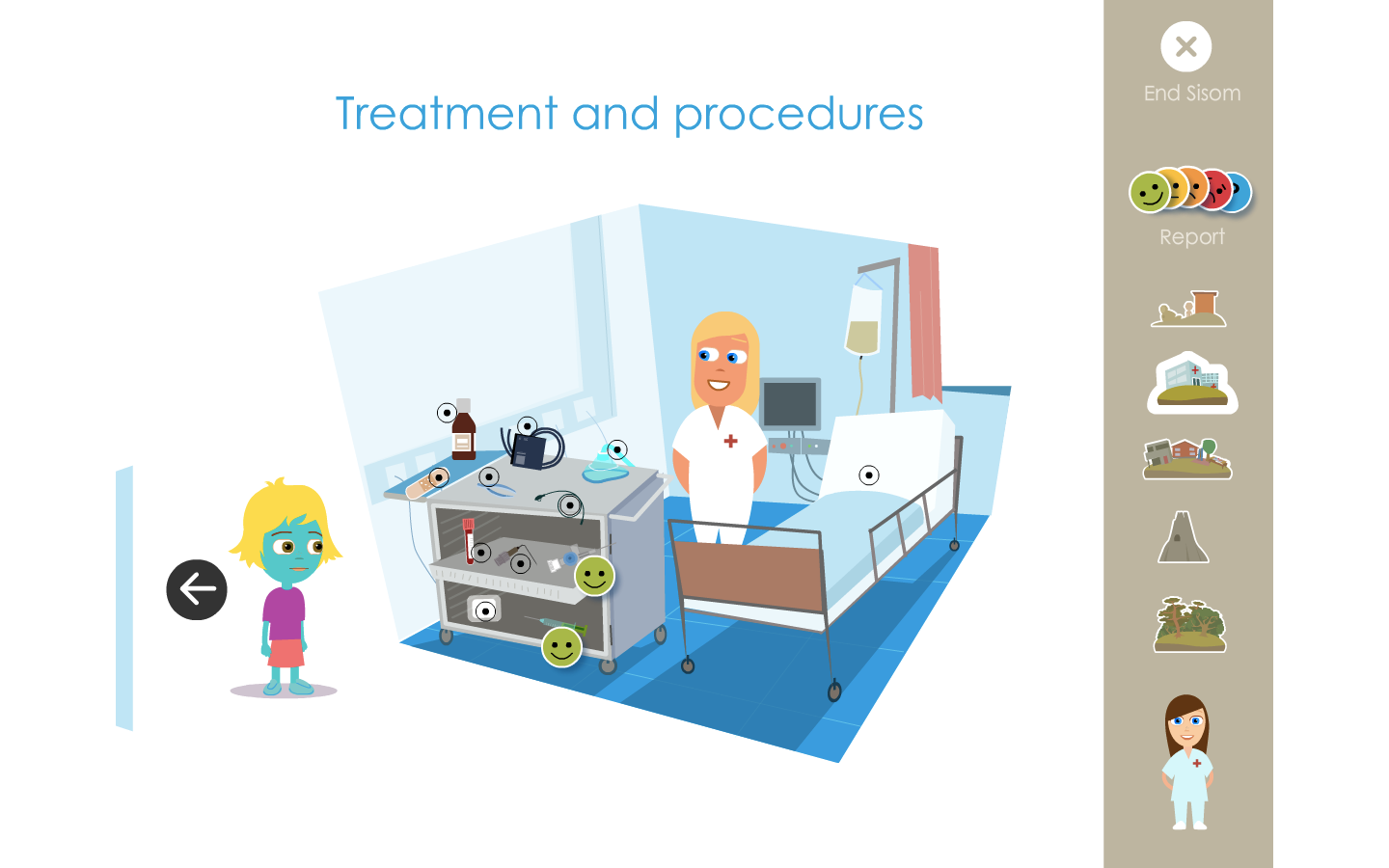 | Relating to Their Condition | Understanding Their Condition |
|  |  | Special Relationship with the Hospital |
|  |  | Difficult Treatments and Procedures |
| 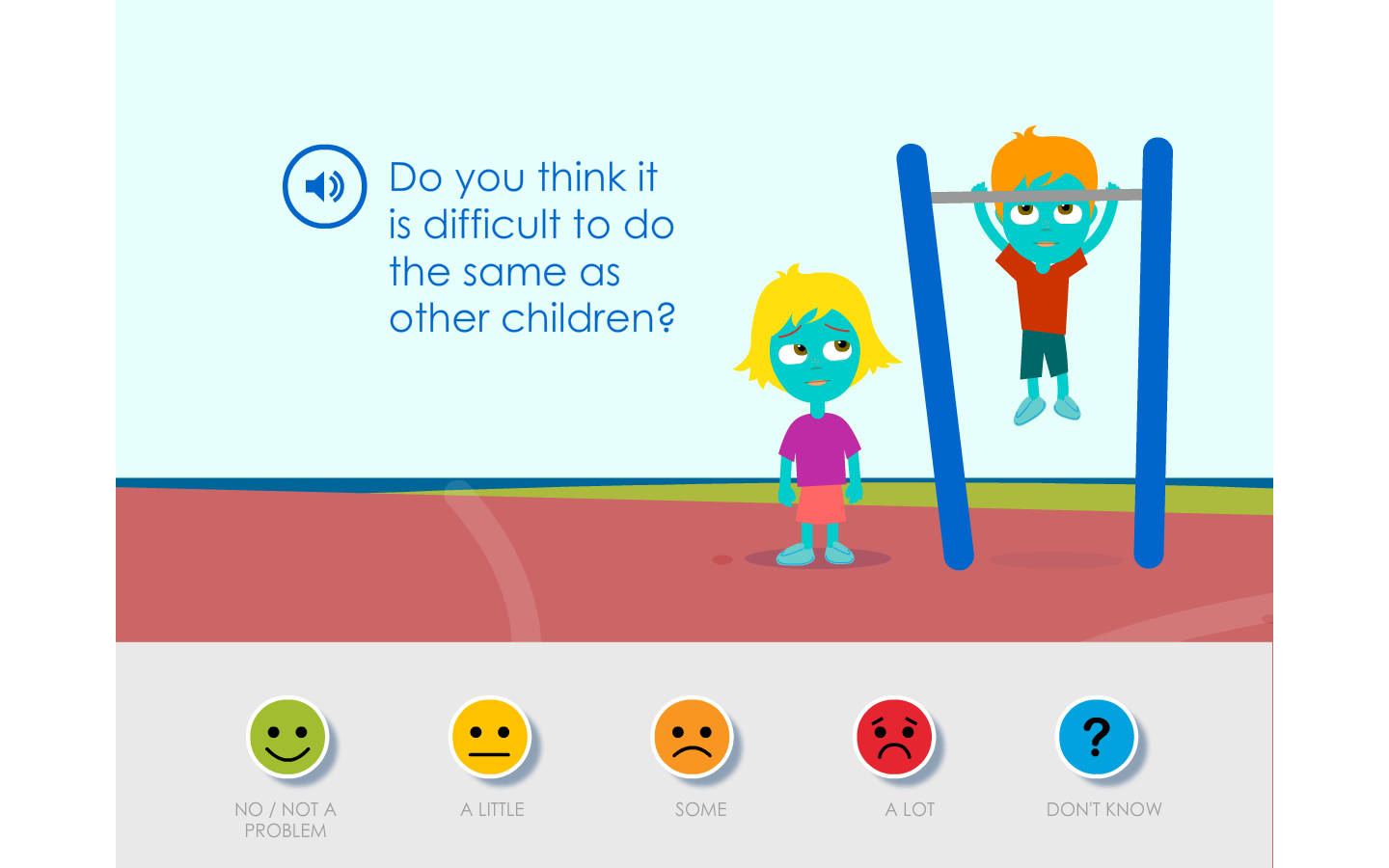 | Reflecting on Capabilities | Perceiving Limitations |
|  |  | Overcoming Isolation |
|  |  | Celebrating Strengths |

^a^OI: osteogenesis imperfecta.
